# Stromal-platelet membrane-inspired nanoparticles (SPIN) for targeted heart repair

**DOI:** 10.1016/j.bioactmat.2025.06.055

**Published:** 2025-07-07

**Authors:** Mingqian He, Yuan Li, Dashuai Zhu, Junlang Li, Meggie Cangu, Panagiotis Tasoudis, Jiazhu Xu, Thomas G. Caranasos, Yi Hong, Ke Huang

**Affiliations:** a Department of Biomedical Engineering, University of North Carolina Chapel Hill and NC State University, North Carolina, USA; b Department of Biomedical Engineering, Columbia University, New York, USA; c Smidt Heart Institute, Cedars-Sinai Medical Center, Los Angeles, CA, USA; d Department of Biological Sciences, North Carolina State University, Raleigh, NC, USA; e Division of Cardiothoracic Surgery, the University of North Carolina at Chapel Hill, Chapel Hill, NC, USA; f Department of Bioengineering, University of Texas at Arlington, Arlington, USA; g Department of Pharmaceutical Sciences, Texas A&M University, Kingsville, USA

**Keywords:** Hybrid membrane coating, Nanoparticles, Cardiac targeting, Heart repair

## Abstract

Myocardial infarction (MI), commonly known as a heart attack, remains a leading cause of death worldwide. Standard treatments, such as coronary stent placement or coronary artery bypass graft surgery, aim to restore blood flow to ischemic myocardial tissue. However, a significant complication of these procedures is ischemia/reperfusion (I/R) injury, which occurs when blood flow is restored, triggering oxidative stress, inflammation, and calcium overload that can further damage the heart. To limit the I/R injury following the coronary recanalization of an MI heart, we designed stromal-platelet membrane-inspired nanoparticles (SPINs) that consist of a poly (lactic-co-glycolic acid) (PLGA) core, decorated by a dual membrane coating: a platelet membrane for precise adhesion to the damaged endothelium area and a stromal cell membrane to enhance receptor-ligand interactions and immune-evasiveness. This unique dual-membrane configuration synergistically reduces fibrosis and inflammation while promoting angiomyogenesis. This combination integrates the vascular injury targeting and immune-evasive properties of the nanoparticle, making this dual-membrane design a promising add-on intervention to augment post- percutaneous coronary intervention recovery, enhancing outcomes and offering potential improved cardiac repair.

## Highlights

• SPIN targets I/R injury after PCI, addressing a clinical gap where no effective current therapies exist.
• SPIN combines platelet and CSC membrane features to enable immune evasion, immunomodulation, and intercellular contact-dependent pro-regenerative signaling.
• SPINs are stable, release of key growth factors over 14 days, and promote cardiomyocyte survival in vitro and in vivo.
• SPINs induces a pro-regenerative immunological shift by downregulate CCL5 and upregulate TIMP-1.

## Graphical abstract

**Figure:**
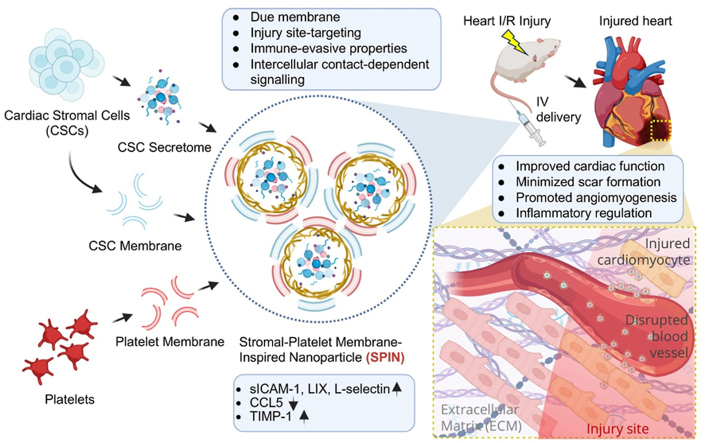

## 1 Introduction

Myocardial infarction (MI) remains a major cause of mortality [1]. This condition commonly arises from coronary artery disease, characterized by the gradual accumulation of atherosclerotic plaques within the coronary arteries. This progressive narrowing and eventual occlusion of the coronary arteries usually requires recanalization through percutaneous coronary intervention (PCI) or coronary artery bypass grafting (CABG) to restore blood flow, which can lead to further ischemic/reperfusion (I/R) injury to the infarcted cardiac tissue *via* rapidly reintroduced oxygen-rich blood [2]. This sudden paradoxical injury further increases the risk of arrhythmias and cell death. Therefore, preventing or mitigating I/R injury can preserve more viable myocardium and improve overall clinical outcomes following PCI or CABG.

Subsequent to cardiac I/R injury, a complex interplay occurs between various cell types, including cardiomyocytes, fibroblasts, and endothelial cells, which collectively contribute to tissue repair processes. The intercommunication between these cells through paracrine signaling [[3], [4], [5]], direct intercellular interactions [6], and interactions with the extracellular matrix [7] plays a crucial role in the healing process. However, dysregulation in these interactions can lead to adverse outcomes, such as excessive fibrosis and ventricular remodeling, and thus ultimately impair cardiac function. To enhance post-I/R repair, cell membrane-coated micro- and nano-sized particles have emerged as promising tools for modulating cellular communication and improving treatment efficacy due to their ability to mimic the biological properties of native cells, thereby enhancing biocompatibility, circulation time, and targeted delivery [[8], [9], [10], [11]]. By leveraging the unique surface markers and functional proteins of specific cell membranes, such as those derived from platelets, stem cells, or macrophages, these biomimetic particles can facilitate immune evasion, promote tissue regeneration, and modulate inflammatory responses [[12], [13], [14], [15], [16], [17]].

Cardiac stromal cells (CSCs) have been extensively researched in cardiac regenerative medicine due to their heart repair potential *via* secreting paracrine factors [[18], [19], [20], [21], [22]], extracellular vesicles [[23], [24], [25], [26]], and membrane-induced cell-cell contact [6]. To harness the benefits of CSC-secretomes without the complexities of live-cell therapy, researchers have turned to poly (lactic-co-glycolic acid) (PLGA) microparticles designed to mimic the therapeutic effects of living cells [9,27]. However, intravenous administration of microparticles increases the risk of aggregation in the bloodstream, potentially obstructing blood flow due to their size and procoagulant properties [28]. Moreover, despite their promising design, PLGA microparticles demonstrate limited heart-targeting capability due to their non-specific biodistribution and rapid clearance, which restricts their effectiveness in delivering therapeutics to the heart *via* a minimally invasive route [29,30].

Nanoparticles hold promise in addressing unmet clinical needs, particularly in cardiovascular disease treatment [31]. It offers unique advantages over traditional drug delivery systems due to its nanoscale size [32], high surface area-to-volume ratio [33]and the ability to be engineered for precise targeting [[34], [35], [36], [37]], which enables them to navigate the bloodstream more efficiently and with greater organ-targeting ability. However, current nanoparticles tend to exhibit non-specific biodistribution after intravenous injection, accumulating in the liver, spleen, and lungs, which reduces the concentration of the therapeutic agent at the target site [38]. Additionally, they are often rapidly cleared from the bloodstream by the reticuloendothelial system, primarily through macrophage uptake in the liver and spleen. This short circulation time also limits the amount of therapeutic agents that can reach the target tissue [39].

To address these limitations, we developed the stromal-platelet membrane-inspired nanoparticles (SPINs). This dual-membrane approach tackles the limitations of non-specific biodistribution and rapid clearance by the immune system *via* the targeting capabilities of platelet membranes to the vascular injury site, along with the microenvironment modulation potential of stromal cell membranes. Additionally, the PLGA core of SPIN enables controlled, sustained release of therapeutic factors where prolonged therapeutic action is required [34,40]. Platelet membranes have a natural ability to adhere to an injured endothelium. Following emergency PCI or CABG procedures, platelets accumulate in the damaged area and become activated, expressing adhesion proteins that facilitate their contribution to I/R injury through microthrombus formation, enhanced platelet–leukocyte aggregation, and the release of potent vasoconstrictor and pro-inflammatory molecules [41,42]. Our previous studies capitalized on this concept and designed various therapeutics decorated with platelet membranes, which greatly improved their targeting efficiency to an MI-injured heart [27,34,43]. In addition to platelet functionality, the inclusion of CSC membranes further enhances SPIN's therapeutic profile by imparting both immunomodulatory and immune-evasive properties. CSCs express MHC class I but lack MHC class II and the costimulatory molecules CD80 and CD86, which are essential for the activation of CD4^+^ T cells and induction of effector lymphocytes [44]. This unique immunophenotype reduces the likelihood of eliciting a strong adaptive immune response, thereby supporting prolonged circulation and reduced clearance of SPINs. Moreover, CSC membranes contribute bioactive surface cues derived from their native regenerative microenvironment and promote cardiomyocyte survival and tissue proliferation [6,45]. Therefore, SPIN is intended as a targeted adjunct therapy to standard reperfusion procedures such as PCI or CABG, specifically to mitigate I/R and post-infarction myocardial damage, for which no effective therapies currently exist.

## 2 Results

### 2.1 Fabrication and characterization of SPINs

**Fig. 1:**
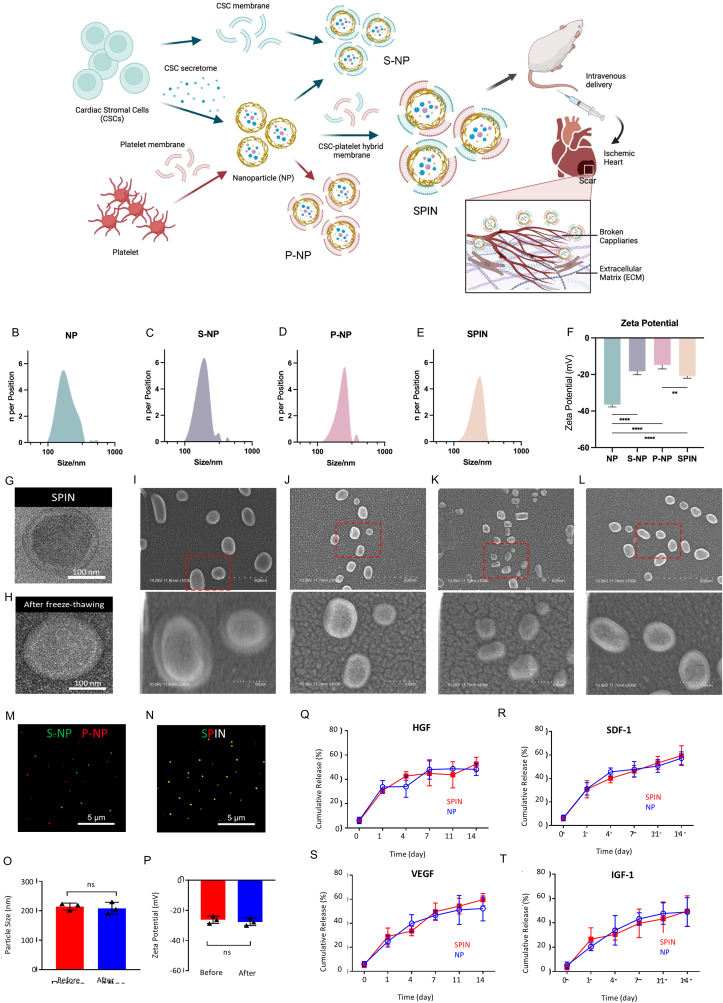
**Fabrication and Characterization of cardiac stromal cell (CSC)/platelet-inspired nanoparticle (SPIN).** (A) Schematic illustration of the fabrication that incorporates both CSC-platelet hybrid membrane and CSC-secreted factors to target the heart after myocardial ischemia/reperfusion (I/R) injury. (B–E) Size distribution of bare nanoparticle (NP), S-NP, P-NP, and SPIN measured by DLS. (F) Zeta potentials of NP, S-NP, P-NP, and SPIN. (G) Transmission electron microscopy (TEM) image showing the ultrastructure of SPIN. (H) TEM image showing the ultrastructure of SPIN after freeze-thawing. (I–L) Scanning electron microscope (SEM) image showing the morphology of air-dried NP, S-NP, P-NP, and SPIN. (M, N) Confocal laser scanning microscopy (CLSM) images of a mixture of PLGA nanoparticles functionalized with CSC membrane (DiO-labeled, green) and secretome (designated S-NP), and PLGA nanoparticles functionalized with platelet membrane (DiI-labeled, red) and secretome (designated P-NP), and SPIN functionalized with a mixture of CSC membrane and platelet membrane and secretome. (O) The comparison of particle size and (P) zeta potential of SPIN before and after freeze-thawing. (Q–T) Enzyme-linked immunosorbent assays of the growth factor release profiles of SPINs. Scale bars, 100 nm. All data are mean ± SD. Comparisons between any two groups were performed using a two-tailed unpaired Student's t-test. Comparisons among more than two groups were performed using one-way ANOVA followed by a post hoc Bonferroni test (n = 3). ns, not significant; ∗*P* < 0.05; ∗∗*P* ≤ 0.01; ∗∗∗*P* ≤ 0.001; ∗∗∗∗*P* ≤ 0.0001.

To synthesize functionalized SPINs, we fabricated a PLGA core encapsulating concentrated CSC secretomes and coated the nanosized core with a blend of CSC and platelet membrane fragments (Fig. 1A). CSC secretome-loaded PLGA core (NP) were either coated with CSC membranes (S-NPs) or platelet membranes (P-NPs). In our study, dynamic light scattering analysis indicated that NP, S-NP, P-NP, and SPIN have an average diameter of 200 ± 35 nm and a narrow size distribution (polydispersity index (PDI) = 0.200) (Fig. 1B–E). After coating with CSC-Platelet hybrid membranes, the zeta potential of SPIN increased by approximately 16 mV compared to NP, reaching a value of −38 mV. The zeta potential of S-NPs was measured at −35 mV, while that of P-NPs was −32 mV (Fig. 1F). This observation aligns with previous studies on platelet membrane-coated nanoparticles, where the low-negativity platelet membranes masked the highly negative inner PLGA core [40], which reduces the overall surface negativity, making the nanoparticle more biocompatible and less prone to rapid clearance by the immune system. Similar to P-NPs, SPINs also partially shield the negative charge of the inner nanoparticle core. Transmission electron microscopy (TEM) studies verified the structure of the SPIN (Fig. 1G). To evaluate the structural stability of SPINs, we compared freshly fabricated SPINs with freeze-thawed SPINs and found no significant morphological changes, indicating that the freeze-thaw process did not affect their structural integrity (Fig. 1G and H). Despite being coated with different cell membranes, the air-dried nanoparticles maintained a consistent three-dimensional morphology across S-NPs, P-NPs, and SPINs, with no significant differences observed (Fig. 1I–L). Moreover, there was no notable aggregation evident as shown in the scanning electron microscope (SEM) images. To further visualize the mixed membrane coating, confocal laser scanning microscopy (CLSM) was used to verify the colocalization of DiI-labeled platelet membranes in P-NPs and DiO-labeled CSC membranes (Fig. 1M and N) in SPIN. The co-expression of red and green labels demonstrated the successful coating of the two cell membrane fragments on the surface of NPs. Additionally, we confirmed that the SPINs maintained a consistent size and surface charge before and after the freeze-thaw process (Fig. 1O and P). To investigate whether the dual-membrane coating affects the controlled release of CSC secretomes from NP core, we analyzed the release profiles of four representative CSC secretome proteins—including hepatocyte growth factor (HGF), stromal cell-derived factor-1 (SDF-1), vascular endothelial growth factor (VEGF), and insulin-like growth factor 1(IGF-1), for 14 days (Fig. 1Q–T). NPs and SPINs displayed no significant differences in protein release ability. These results indicated that SPINs were successfully fabricated by encapsulating CSC secretomes within a PLGA core and coating it with dual-membranes, maintaining uniform size, structural integrity, and surface charge while preserving controlled protein release ability.

### 2.2 Regenerative potency of SPINs *in vitro*

We evaluated the cytocompatibility of S-NPs, P-NPs, and SPINs by assessing their effects on H9c2 cardiomyocytes through cell viability assays. H9c2 cells co-cultured with S-NPs, P-NPs, and SPINs exhibited high survival rates from 24 to 48 h post-treatment, independent of nanoparticle concentration (Fig. S1A). Notably, cells treated with SPINs demonstrated significantly greater proliferative potential compared to those treated with S-NPs or P-NPs (Figure S1 B). In conclusion, these findings indicate the excellent biocompatibility and biosafety of SPINs, as well as their enhanced *in vitro* regenerative potential compared to platelet membrane-coated nanoparticles. This enhancement may be attributed to specific interactions between the stromal cell membrane on the nanoparticles and injured cardiomyocytes, leading to the activation of regenerative β1 integrin signaling [6].

### 2.3 Retention and biodistribution of SPINs in a mouse model of I/R

**Fig. 2:**
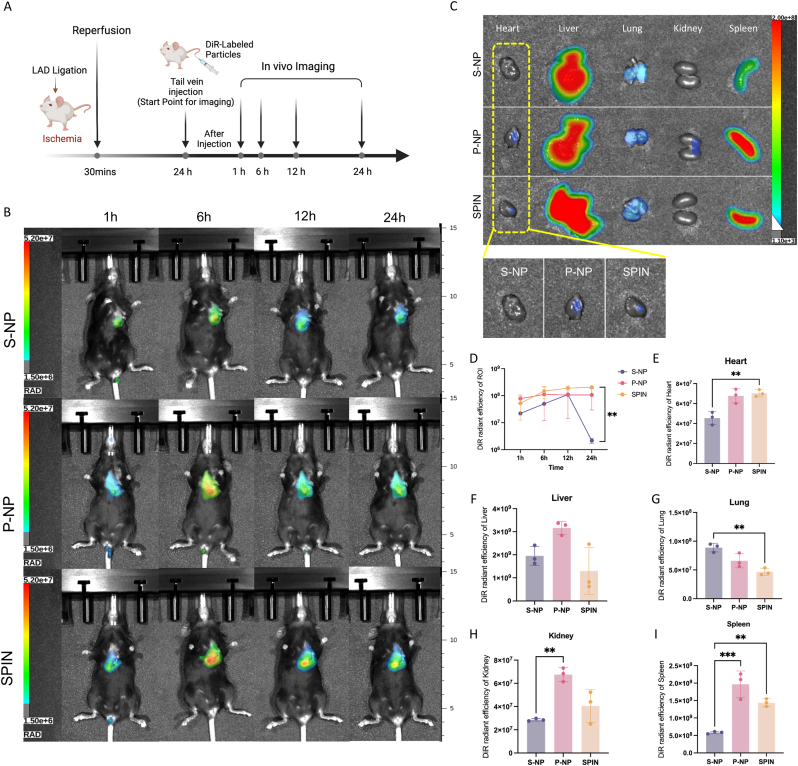
***In vivo* retention and biodistribution after SPINs administration.** (A) Schematic study design of biodistribution in the C57BL/6 mice model of myocardial I/R injury. (B) Representative *in vivo* imaging *via* the Spectrum Instruments Imaging system of biodistributions of S-NPs, P-NPs, and SPINs over time after intravenous delivery. (C) Representative *ex vivo* fluorescent imaging of mouse organs (heart, liver, lung, kidney, and spleen) at 24 h post-intravenous delivery of S-NPs, P-NPs, and SPINs. (D) Quantitative analysis *via* Aura imaging software of radiant efficiency for *in vivo* imaging over time post-intravenous delivery. (E–I) Quantitative analysis of radiant efficiency of heart, liver, lung, kidney, and spleen. All data are mean ± SD. P values are determined by two-way ANOVA analysis (D) and one-way ANOVA analysis followed by post hoc Bonferroni test (E–I) (n = 3); ns, not significant; ∗*P* < 0.05; ∗∗*P* ≤ 0.01; ∗∗∗*P* ≤ 0.001; ∗∗∗∗*P* ≤ 0.0001.

This study also aimed to assess the heart-targeting capability of SPINs in a C57BL/6 mouse model of I/R injury, which features a relatively thinner chest wall compared to rats, facilitating live imaging. To induce ischemic injury, mice underwent temporary ligation of the left anterior descending (LAD) coronary artery for 30 min, followed by reperfusion. After 24 h, mice were randomly assigned to three groups and received intravenous tail vein injections of DiR-labeled S-NPs, P-NPs, or SPINs, respectively (Fig. 2A). IVIS imaging was performed at 1 h, 6 h, 12 h, and 24 h after the tail vein injection (Fig. 2 B), and then the major organs were harvested for *ex vivo* fluorescent imaging (Fig. 2 C). From the quantitative analysis of fluorescent signals of the heart region, the P-NPs and SPINs group showed significantly better retention after 24 h (Fig. 2D). The quantitative region-of-interest (ROI) analysis *via* radiance efficiency, which is a measure of photon flux of the fluorescent-labeled NPs in the organ of interest *ex vivo*, confirmed that the infarcted hearts that received SPINs and P-NPs exhibited a significantly higher fluorescent signal in the hearts than those that received S-NPs (Fig. 2E–I). IVIS quantification of *ex vivo* organs revealed that the majority of nanoparticles accumulated in the liver and lungs, while those coated with platelet membranes exhibited an increased propensity for heart retention and possible renal clearance. These IVIS imaging and *ex vivo* analysis demonstrated that SPINs and P-NPs significantly enhanced their heart retention in a mouse model of I/R injury, while most nanoparticles may be cleared by the liver and lungs, with platelet membrane coating potentially facilitating renal clearance.

### 2.4 SPIN treatment effects in a rat model of I/R

**Fig. 3:**
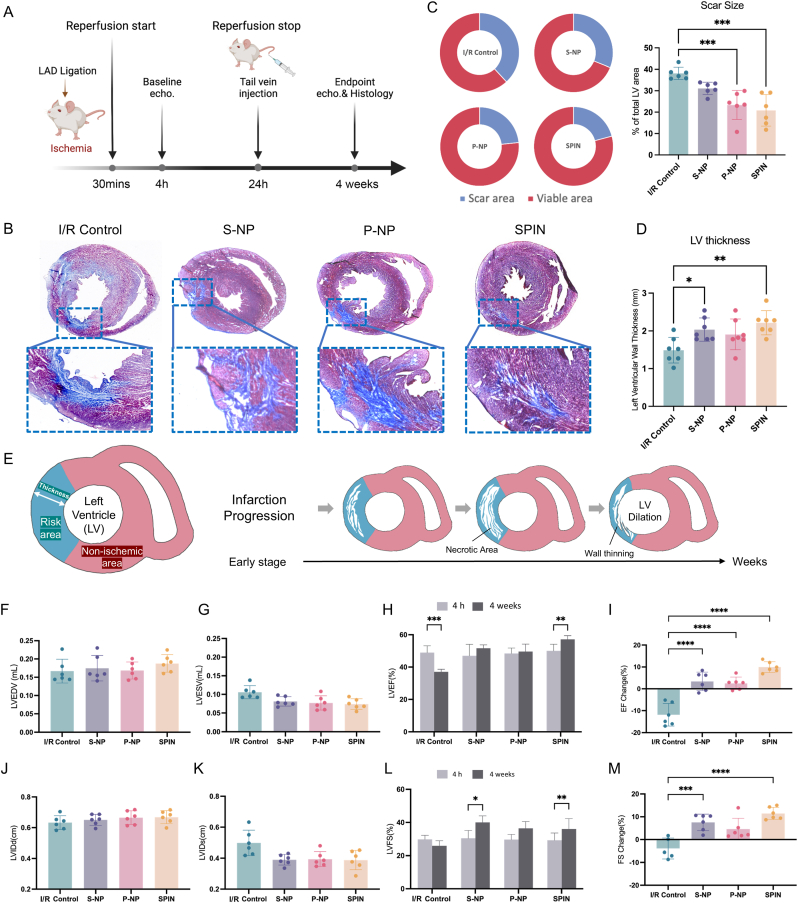
**Therapeutic benefits of SPIN treatment in the rat model of I/R.** (A) Study design of SPIN therapy in a rat model of myocardial I/R injury. (B) Heart morphology imaging by Masson's trichrome staining (blue: scar tissue; red: viable myocardium) (C) Comparison of scar size portion means between groups and quantification of scar size in total left ventricle area. (D) Quantification of left ventricular thickness. (E) Schematic image of left ventricle remodeling after myocardial infarction. (F, G) Left ventricular end-diastolic (F) and end-systolic (G) volumes (LVEDV and LVESV) were measured at 4 weeks after different treatments. (H) Left ventricular ejection fraction (LVEF) at baseline (4 h post-I/R) and 4 weeks afterward. (I) Treatment effects were assessed by the change in LVEF over the 4-week time course relative to baseline. (J, K) Left ventricular end-diastolic (I) and end-systolic (J) diameters (LVIDd and LVISd) were measured at 4 weeks after different treatments. (L) Left ventricular ejection fraction (LVFS) at baseline (4 h post-I/R) and 4 weeks afterward. (M) Treatment effects were assessed by the change in LVFS over the 4-week time course relative to baseline. P values are determined by one-way ANOVA followed by a post hoc Bonferroni test. (n = 6); ns, not significant; ∗*P* < 0.05; ∗∗*P* ≤ 0.01; ∗∗∗*P* ≤ 0.001.

The rat model of I/R offers a larger heart size compared to mice, allowing for more precise surgical interventions, including LAD ligation and reperfusion. This facilitates more reliable assessments of cardiac function, histological analysis, and biomaterial delivery. Therefore, we tested the biological activity of SPINs in the Sprague-Dawley (SD) rat model of I/R injury (Fig. 3A). Following a 30-min temporary ligation of the LAD to induce ischemic injury of the left ventricle wall, all rats underwent 24 h of reperfusion before being randomly assigned to four groups. Each group received either saline (I/R control), S-NPs, P-NPs, or SPINs *via* tail vein injection. After four weeks, all rats were euthanized, and their hearts were collected for Masson's trichrome staining (Fig. 3B). Quantitative analysis demonstrated that treatment with P-NPs and SPINs resulted in the smallest scar size (Fig. 3C), while the SPIN-treated group exhibited the highest preservation of viable myocardium within the injury region (Fig. S2). Additionally, left ventricular wall thickness was maintained in both the S-NP and SPIN-treated groups (Fig. 3D). These results demonstrated a protective effect of SPINs on cardiac morphology. This structural preservation is critical, as infarction-induced ventricular dilation typically leads to wall thinning and weakening, ultimately impairing left ventricular function and reducing cardiac output (Fig. 3E). We also conducted echocardiography of heart function at 4 h post-I/R injury and 4 weeks after treatment (Fig. 3F–M). After 4 weeks, the left ventricular ejection fraction (LVEF) was significantly reduced in the saline-treated group, whereas in P-NPs and S-NPs-treated groups, LVEF remained unchanged. LVEF in the hearts of the SPIN-treated group showed a more significant increase. The beneficial effects on cardiac function in P-NPs, S-NPs, and SPIN-treated rats compared to saline control were demonstrated by a reduction of left ventricular end-systolic volume (LVESV). In contrast, there was no significant difference in left ventricular end-diastolic volume (LVEDV). Measurements of left ventricular internal diameter at end-diastole (LVIDd), left ventricular diameter at end-systole (LVIDs), and left ventricular fractional shortening (LVFS) supported these conclusions and demonstrated a better pumping ability in the SPIN-treated group (Fig. 3J–M). Altogether, the *in vivo* study in a rat model of I/R injury demonstrated that SPIN treatment preserved left ventricular function, minimized scar formation, and maintained myocardial structure compared to other control groups, with echocardiographic analysis confirming improved LVEF by reduced LVESV four weeks post-treatment.

### 2.5 SPIN therapy promotes angiomyogenesis in a rat model of I/R injury

**Fig. 4:**
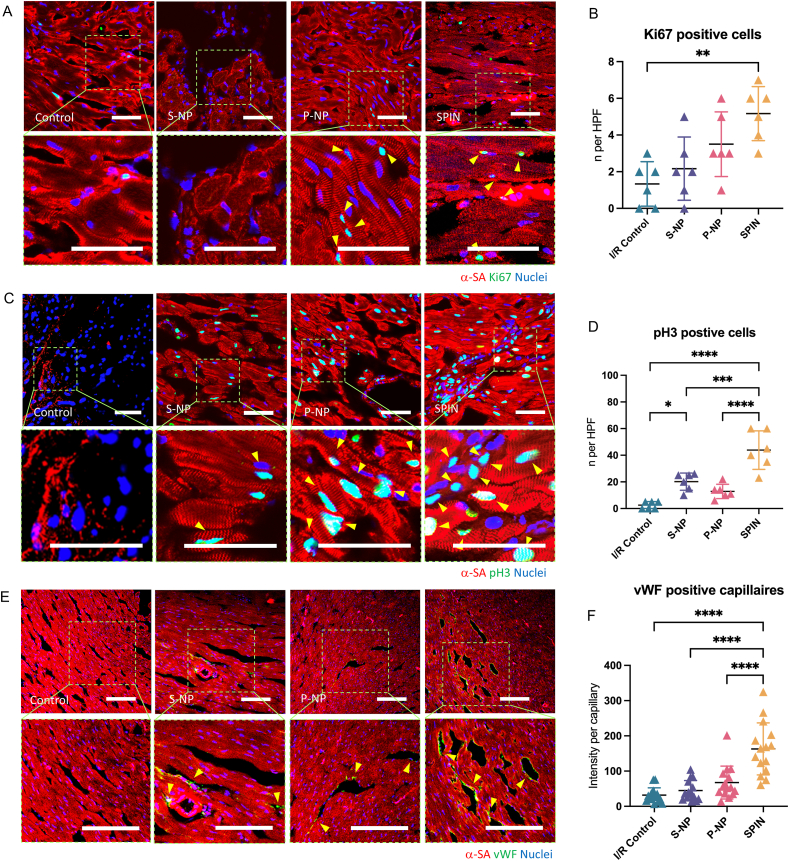
***In vivo* bioactivity of SPINs.** (A) Representative images showing the cardiomyocytes in late G2/mitosis phase as indicated by α-SA and phospho-histone H3 (pH3) double-positive staining in the peri-infarct regions of the hearts 4 weeks after injection with saline, P-NPs, S-NPs, and SPIN. (B) Quantification of pH3-positive cardiomyocytes at week 4 in different treatment groups. (n = 6) (C) Images showing the proliferation of cardiomyocytes by α-SA and Ki67 double-positive staining. (D) Quantification of Ki67-positive cardiomyocytes at week 4. (n = 6) (E) Images showing the vascularization of capillary blood vessels by vWF staining. (F) Quantification of vWF-positive per capillary at week 4. (n = 15). All data are means ± standard deviation (SD). Comparisons among groups were performed using one-way ANOVA followed by post hoc Bonferroni test; ns, not significant; ∗*P* < 0.05; ∗∗*P* ≤ 0.01; ∗∗∗*P* ≤ 0.001; ∗∗∗∗*P* ≤ 0.0001. Scale bars, 50 μm.

To assess the therapeutic potential of SPINs in myocardial repair, immunostaining analysis was performed on heart samples collected four weeks post-treatment. Cardiomyocyte proliferation was evaluated through the costaining of cardiomyocyte structural protein α-sarcomeric actinin (α-SA) and a well-established marker of cellular proliferation-Ki67 (Fig. 4A). Quantitative analysis revealed a significantly higher number of Ki67-positive cardiomyocytes in the peri-infarct region of hearts treated with P-NPs and SPINs compared to those in the saline-injected and S-NP-treated groups (Fig. 4B), suggesting that these nanoparticles, particularly SPINs, promoted cardiomyocyte proliferation in the injured myocardium. To further investigate cell-cycle activity, phosphorylated histone H3 (pH3), a mitotic marker specific to late G2/M phase, was assessed (Fig. 4C and D). The results demonstrated that SPIN treatment markedly enhanced cardiomyocyte proliferation within the periinfarcted region. In addition, myocardial vascularization was evaluated by staining for von Willebrand factor (vWF), an endothelial cell marker (Fig. 4E and F). Nkx2.5, a transcription factor crucial for early vessel development, and Flk-1, a primary receptor for vascular endothelial growth factor (VEGF) (Fig. S3). Quantitative fluorescence analysis showed enhanced recruitment of endothelial progenitor cells and improved neovascularization following SPIN administration, revealing the angiogenic potential of SPINs in the peri-infarct vasculature. Therefore, these data collectively supported that SPIN treatment significantly promoted angiomyogenesis in the infarcted myocardium.

### 2.6 Immune cell recruitment and inflammatory regulation after SPIN treatment

**Fig. 5:**
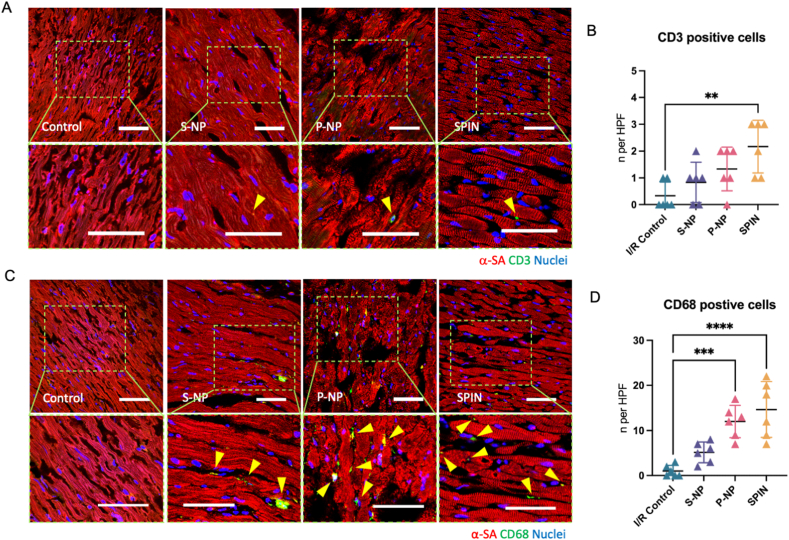
***In vivo* immune cell recreation 4 weeks post-SPIN therapy.** (A) Representative images showing T cell activities *via* staining CD3-positive cells in the peri-infarct regions of the hearts 4 weeks after injection with saline, P-NPs, S-NPs, and SPIN. (B) Quantification of CD3-positive cells at week 4 in different treatment groups. (C) Images showing the macrophage activities *via* staining CD68-positive cells in the peri-infarcted area. (D) Quantification of CD68-positive cells at week 4. P values are determined by one-way ANOVA followed by post hoc Bonferroni test (n = 6); ns, not significant; ∗*P* < 0.05; ∗∗*P* ≤ 0.01; ∗∗∗*P* ≤ 0.001; ∗∗∗∗*P* ≤ 0.0001. Scale bars, 50 μm.

To assess the immunomodulatory effects of SPIN treatment, we performed immunostaining for CD3 and CD68 which are markers of T cells and macrophages, respectively on heart tissues collected four weeks post-treatment. These immune cell populations play crucial roles in post-infarction remodeling by modulating inflammation and tissue repair. At four weeks post-treatment, quantitative analysis demonstrated a significantly higher density of CD3^+^ T cells and CD68^+^ macrophages in the SPIN-treated group compared to saline, S-NP, and P-NP groups (Fig. 5A–D). This data represented a robust and sustained immune response during the transition from the acute inflammatory phase to the chronic reparative phase, where immune modulation is critical for long-term cardiac recovery.

**Fig. 6:**
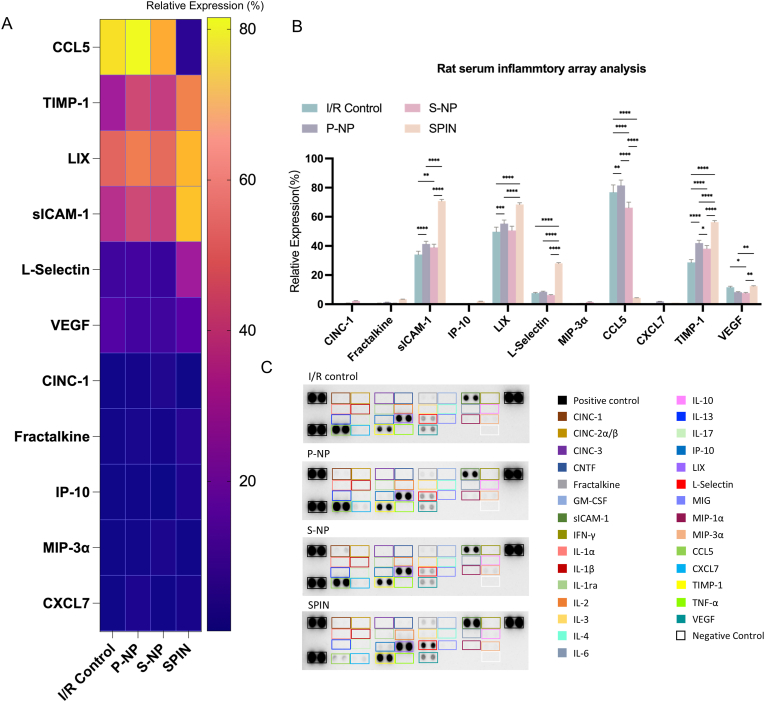
**Inflammation array assay of serum collected from rats.** (A) The heat map of cytokine expression. (B) Quantification of the relative expression rate of cytokines in the array for 4 treatment groups. (C) Images of the cytokines array assay. P values are determined by two-way ANOVA followed by post hoc Bonferroni test (n = 3; 3 times repeated analysis); ns, not significant; ∗*P* < 0.05; ∗∗*P* ≤ 0.01; ∗∗∗*P* ≤ 0.001; ∗∗∗∗*P* ≤ 0.0001.

To further investigate the inflammatory response, we analyzed serum cytokine levels four weeks post-treatment (Fig. 6A and B). The SPIN-treated group exhibited elevated levels of sICAM-1, LIX, and L-selectin, indicative of active immune cell recruitment and endothelial activation. However, CCL5 expression was significantly lower in the SPIN group compared to controls (p < 0.05), suggesting a shift away from chronic inflammation. The downregulation of CCL5 in SPIN-treated hearts suggested a controlled immune response that prevents long-term inflammatory damage while allowing for tissue remodeling. Interestingly, CCL5 inhibition has been reported to exert cardioprotective effects during myocardial reperfusion [81]. Additionally, TIMP-1 expression was significantly upregulated in the SPIN group (p < 0.01), indicating enhanced regulation of extracellular matrix integrity (Fig. 6C). The increased TIMP-1 expression in SPIN-treated hearts suggested a protective effect against fibrosis progression, supporting myocardial structural and functional preservation[82]. Ultimately, these findings highlighted the therapeutic potential of SPINs in modulating inflammation and fostering cardiac tissue repair.

## 3 Conclusion and discussion

MI remains a major contributor to morbidity and mortality worldwide, with ischemia-reperfusion (I/R) injury further aggravating myocardial damage following reperfusion therapy [46]. While restoring blood flow is essential to prevent irreversible cardiomyocyte death, the sudden influx of oxygen initiates oxidative stress, inflammatory cascades, and endothelial dysfunction [47]. This secondary wave of injury extends beyond the infarcted myocardium, exacerbating ventricular remodeling and increasing the risk of heart failure and multi-organ dysfunction. Various cardioprotective strategies, such as ischemic preconditioning, post-conditioning, and therapeutic hypothermia, have been investigated to mitigate I/R injury [48]. However, their clinical application remains limited by logistical constraints, inconsistent outcomes across patient populations, and the lack of universally effective interventions.

Efforts to enhance myocardial repair have primarily centered on two approaches: invasive biomaterial-based scaffolds and systemic biologic therapies. Engineered cardiac patches and biocompatible scaffolds provide structural reinforcement and localized drug delivery. However, these treatments need surgical implantation, which restricts their clinical feasibility [[49], [50], [51], [52], [53], [54], [55]]. On the other hand, systemic delivery methods, including intravenous injection of extracellular vesicles (EVs), viral vectors, liposomes, and polymeric nanoparticles, offer ease of administration but suffer from rapid systemic clearance, non-specific biodistribution, and inefficient myocardial retention. [[56], [57], [58], [59], [60], [61], [62]]. These challenges highlight the need for a targeted, biomimetic, and efficient drug delivery system capable of localizing therapeutic agents to the infarcted myocardium while avoiding off-target accumulation.

While previous membrane-coated nanoparticle platforms have made important advances in targeted delivery and immune modulation [[63], [64], [65], [66]], SPIN builds on these efforts with a hybrid membrane design. This design improves cardiac homing, supports immune evasion, and presents surface signals through cell-cell contact [6] and paracrine factors that promote cardiomyocyte survival, angiogenesis, and immune regulation. Unlike earlier hybrid systems such as platelet and red blood cell composites or platelet and macrophage composites, which mainly aim to extend circulation or reduce immune clearance, SPIN is tailored to achieve both targeted delivery and therapeutic regeneration in the injured heart as a novel infarct-targeting therapeutic platform that integrates platelet-mediated cardiac homing with stromal cell-derived heart benefits. Platelet membranes provide natural adhesion to injured endothelium, enhancing myocardial specificity, while CSC membranes offer cell-cell contact and immune-evasiveness. Our findings indicate that SPIN therapy improves cardiomyocyte proliferation, enhances vascular regeneration, modulates immune responses to prevent chronic inflammation, and preserves cardiac function while reducing maladaptive remodeling. These combined effects highlight SPINs as a multi-functional strategy for post-MI repair, addressing the limitations of existing systemic and scaffold-based therapies. Although hybrid membrane-coated nanoparticles have been explored in other biomedical contexts, such as platelet-RBC hybrid membranes for immune evasion [67], platelet-macrophage hybrids for inflammation targeting [68], and platelet-neutrophil hybrids for cancer therapy [69], there is no previous study that has incorporated CSC membranes into a hybrid nanoparticle system specifically designed for cardiac repair. By merging platelet membrane-derived injury targeting with CSC membrane-derived immune-evasiveness, SPINs represent a new paradigm in myocardial therapy.

Beyond MI, the platelet-stromal cell hybrid membrane technology developed in this study may be adapted to treat other ischemic conditions [36,[70], [71], [72], [73]] that require targeted immune modulation and regenerative intervention. For example, SPINs may be used in organ transplantation to mitigate ischemia-reperfusion injury and improve graft survival. Expanding SPIN therapy to these broader applications would further reinforce its potential as a versatile nanomedicine platform for ischemic tissue repair.

While our study provides compelling evidence supporting the therapeutic potential of SPINs for targeted myocardial repair, several limitations should be acknowledged and addressed in future investigations. First, although the sustained release of stromal cell-derived paracrine factors from SPINs likely contributes to attenuation of oxidative stress, inflammation, and calcium overload [74,75], the precise molecular signaling pathways involved—such as PI3K-Akt, STAT3, or β1 integrin-mediated mechanisms—were not directly assessed in the current study. Second, while our study standardized total membrane input across groups, we did not quantitatively assess the specific membrane composition on SPINs. So, more in-depth mechanistic studies are needed to determine whether these membrane effects are synergistic or additive. Additionally, while our immunostaining and serum cytokine profiling indicate a favorable immune modulation and no apparent systemic toxicity, comprehensive immune profiling, histopathological evaluation of major organs, and long-term safety studies are necessary to fully characterize the immunological behavior of SPINs before clinical translation. Also, the heterogeneity of membrane coating and its impact on cellular uptake is uncertain [76]. Previous studies have shown that membrane integrity influences nanoparticle internalization and target efficiency, yet SPINs are currently fabricated using fragmented membranes, raising concerns about variability in coating efficiency. Moreover, a proportion of nanoparticles still accumulate in the liver, likely due to mononuclear phagocyte system (MPS) clearance. Strategies such as PEGylation, ligand modification, or charge-based engineering may further optimize circulation time and cardiac selectivity [77]. Lastly, we recognize the challenges associated with large-scale production, membrane source standardization, and regulatory classification of hybrid biomimetic nanomedicines. As we advance this platform, future studies will aim to incorporate human-derived membranes, enhance manufacturing consistency, and assess pharmacokinetics and biodistribution in higher-order models to support translational potential. Future research should focus on refining membrane fusion techniques and characterizing membrane protein retention to ensure consistency and functionality.

Despite these challenges, this study introduces a novel and transformative strategy for cardiac repair through the development of SPIN, the first dual-membrane nanoparticle system incorporating CSC membranes. SPIN is distinct from previous platforms as it is fully acellular and purposefully designed to integrate targeting, immune modulation, and regenerative potential in a single system. What makes SPIN particularly novel is its use of CSC membranes, which have not been previously employed in dual membrane nanoparticle platforms. This dual membrane configuration enables SPIN to address the limitations of earlier hybrid membrane systems, which were primarily designed to prolong circulation without offering tissue-specific regenerative functions or meaningful cell-cell interaction, and with limited immune evasion capability. Therefore, SPIN therapy represents a compelling new platform for post-infarction treatment, offering a unique combination of injury targeting, immune evasion, and proreparative signaling. While the early results are promising, the limitations and challenges associated with SPIN, such as large-scale production, long-term safety, and translational applicability, still require further investigation. Nevertheless, we believe that SPIN holds significant potential to complement existing reperfusion strategies and help fill the critical gap in targeted myocardial protection.

## 4 Methods and materials

Materials: All chemical reagents were purchased from Sigma–Aldrich or Thermo Fisher Scientific and were used as received unless specifically noted.

### 4.1 Derivation and culture of rat CSCs

We obtained rat CSCs from the hearts of SD rats as described in previous studies [78,79]. Briefly, hearts were minced into fragments less than 2 mm^3^, followed by digestion with collagenase and then seeded onto fibronectin-coated plates to create explant-derived cells. In about 14 days, explant-derived cells were harvested and seeded in ultralow attachment flasks (Corning Life Sciences, Durham, NC) and grown in suspended culture for cardio-sphere formation. CSCs were obtained by replating cardiospheres on fibronectin-coated plates.

### 4.2 Isolation of platelet membrane

The platelet membrane was isolated from human platelet-rich plasma (PRP, ZenBio, USA) through gradient centrifugation as previously described with modification [51]. Briefly, the PRP was centrifuged at 100×*g* for 20 min. PBS (1 × , pH 7.4) containing 1 mM of EDTA and 2 mM of prostaglandin E1(PGE1) was added to keep platelets inactivated. The isolated platelets were then pelleted by centrifugation at 800×*g* for 20 min at room temperature. The platelet membrane was obtained by three freeze-thaw cycles, followed by sonication. The protein content in the purified platelet membrane was determined by the BCA protein assay for further preparation of the P-NPs group.

### 4.3 Isolation of CSCs membrane

The acquired CSCs were suspended in PBS and combined with Halt protease inhibitors (Thermo Fisher Scientific, MA, USA). The suspension was divided into 1 mL aliquots and stored at −80 °C until use. To generate cell membrane fragments of CSCs, the aliquots were put through freeze-thaw cycles. Subsequently, the samples were washed with PBS and mixed with protease inhibitors before undergoing sonication. The protein content in the purified cardiac stromal cells membrane was determined by the BCA protein assay for further preparation of the S-NPs group. The size distribution and morphology of the CSC membranes were assessed using NanoSight (Malvern) and transmission electron microscopy.

### 4.4 Preparation of CSC secretome-loaded PLGA nanoparticles

To harvest conditioned media, the CSCs were cultured in serum-free media for 14 days, and the supernatant was collected. Conditioned media were concentrated by lyophilization and reconstitution. Briefly, conditioned media were collected and filtered through a 0.22 μm filter into a sterile 15 or 50 mL conical, as appropriate. The filtered conditioned media was then stored at −80 °C for at least 24 h or until solid and then lyophilized by a LABCONCO FreeZone 2.5 L Freeze Dry System. CSC-secretome-loaded PLGA nanoparticles (NPs) were fabricated by a water/oil/water (w/o/w) emulsion technique. Briefly, the conditioned media, as the internal aqueous phase with polyvinyl alcohol (PVA) (0.1 % w/v), were mixed in methylene chloride (DCM) containing PLGA as the oil phase. The mixture was then sonicated on ice for 30s using a sonicator (Misonix, XL2020, Farmingdale, NY, USA). Subsequently, the primary emulsion was immediately introduced into water with PVA (0.7 % w/v) to produce a w1/o/w2 emulsion. The secondary emulsion was emulsified for 5 min on a high-speed homogenizer. The w1/o/w2 emulsion was continuously stirred overnight at room temperature to promote solvent evaporation. To determine the secretome loading capacity and efficiency, the NPs were pelleted by centrifugation at 20,000 g, and the non-encapsulated amount of secretome in the supernatant was measured using a BCA protein assay.

### 4.5 Fabrication and Characterization of P-NPs, S-NPs, SPINs

0.5 mL of membrane solution containing 0.36 mg membrane protein was mixed with 0.5 mL of NPs (5 × 10^9^ particles mL^−1^) to fabricate the membrane-coated nanoparticles by ultrasonication for 5 min. After pelleting the membrane-coated nanoparticles from the remaining free hybrid membrane by centrifugation at 20,000×*g*, the amount of membrane protein in the remaining solution was measured using a BCA protein assay. The membrane coating efficiency was determined by comparing the difference between the amount of total membrane protein before the membrane coating and that of the remaining free membrane. The pelleted nanoparticles were then resuspended back to their original volume, followed by examination using NanoSight NS300 to determine the total number of membrane-coated nanoparticles for calculating the amount of membrane protein coated onto each NP.

### 4.6 Physicochemical characterization

Nanoparticle size and surface zeta potential were determined using dynamic light scattering (DLS) with a Malvern ZEN 3600 Zetasizer. The concentration of nanoparticles was analyzed using a NanoSight NS300. The nanoparticle structure was visualized through a JEOL JEM-2000FX transmission electron microscope following negative staining with vanadium (Abcam). To evaluate the stability of various nanoformulations over time, bare NCs, P-NPs, S-NPs, and SPINs were suspended in PBS (1 × , pH 7.4) at a concentration of 1 × 10^9^ particles mL^−1^. Changes in particle size were assessed using DLS at pre-determined time points over two weeks. To investigate stability in serum, the nanoformulations were incubated with 50 % fetal bovine serum (Hyclone, USA), and changes in particle sizes within 4 h were measured using DLS. Long-term stability was determined by examining particle size changes using DLS before lyophilization in 10 wt% sucrose and after resuspension in PBS (1 × , pH 7.4) back to the original volume.

### 4.7 Growth factor release study

Total protein and growth factor releases from SPINs were assessed as previously described [9,80]. Briefly, freeze-dried SPINs were dissolved in DCM, followed by the addition of PBS to the solution. The sample was then vortexed for 5 min, allowing proteins to transfer from the oil phase to the water phase. After centrifugation, the protein concentration in the water phase was measured using a BCA protein assay. For growth factor release studies, nanoparticles were incubated in PBS at 37 °C. Supernatant was collected at various time points (day 3, 7, 11, and 14) after centrifugation at 20,000×*g* for 30 min to pellet the nanoparticles. Concentrations of different growth factors were determined using ELISA kits (R&D Systems, USA) according to the manufacturer's instructions. The data were averaged from three independent measurements.

### 4.8 Cell viability and proliferation assay

Embryonic rat heart-derived H9C2 cardiomyoblasts (Sigma-Aldrich) were exposed to various SPIN concentrations of SPINs, with PBS (1X, pH 7.4) as a control. Cell viability and proliferation were evaluated using a Cell Counting Kit-8 (CCK-8) following the manufacturer's guidelines.

### 4.9 *In vivo* biodistribution

All animal work was compliant with the Institutional Animal Care and Use Committee (IACUC) of the University of North Carolina at Chapel Hill and North Carolina State University. Briefly, female C57BL/6 mice (10 weeks old, The Jackson Laboratories) were anesthetized with 1.5 % isoflurane. Under sterile conditions, the heart was exposed by a left thoracotomy, and a 30-min ischemia was achieved by temporal ligation of the LAD coronary artery. 24 hrs later, the mice I/R injury model was created, animals were randomized to receive intravenous injection of DiR-labeled S-NPs (dose: 1 × 10^8^ particles/mouse), P-NPs (dose: 1 × 10^8^ particles/mouse), SPINs (dose: 1 × 10^8^ particles/mouse). *In vivo* imaging was performed 1, 6, 12, and 24 h after the injection by the Spectrum Instruments Imaging system. The mice were euthanized and then autopsied to collect major organs for *ex vivo* fluorescent imaging. The images were analyzed by Aura imaging software. All background radiance was removed based on images of mice injected with non-fluorescent-labeled NPs as a reference. Quantification bar graphs were generated *via* Prism 9.

### 4.10 Rat model of myocardial I/R injury

Briefly, 5–7 weeks old female SD rats (5–7 weeks old, Charles River Laboratories) were anesthetized with 1.5 % isoflurane, and were given intraperitoneal administration of anesthetic combination (0.8–0.9 μ l/g; xylazine and ketamine with proportion 1:2). Under sterile conditions, the heart was exposed by a left thoracotomy, and a 30-min ischemia was achieved by temporal ligation of the LAD coronary artery. After 24 h of reperfusion, animals were randomized to receive intravenous injection of S-NPs (dose: 5× 10^9^ particles kg^−1^ mouse), P-NPs (dose: 5 × 10^9^ particles kg^−1^ mouse), SPINs (dose: 5 × 10^9^ particles kg^−1^ mouse), and saline (control) every 7 days for 4 weeks, respectively. There were a total of four injections in 4 weeks.

### 4.11 Cardiac function assessment

All animals underwent transthoracic echocardiography while anesthetized at 4 h post-ischemia/reperfusion (I/R) injury and 4 weeks after treatment, using a VisualSonics Vevo 2100 Imaging System. The hearts were imaged in two-dimensional long-axis views at the point of maximum left ventricular diameter. Measurements of left ventricular end-diastolic volume (LVEDV), left ventricular end-systolic volume (LVESV), left ventricular internal diameter in diastole (LVIDd), and left ventricular internal diameter in systole (LVIDs) were taken. Left ventricular ejection fraction (LVEF) and left ventricular fractional shortening (LVFS) were determined by measurements taken from the infarcted area. All measurements were performed in a random order, with both the surgeon and echocardiographer being unaware of the treatment groups. Quantification bar graphs were generated *via* Prism 9.

### 4.12 Heart morphometry

Hearts were harvested and sliced into 5 μm-thick tissue sections. Masson's trichrome staining was performed, and images were captured using a PathScan Enabler IV slide scanner (Advanced Imaging Concepts, USA). Image analysis was conducted using NIH ImageJ software, focusing on viable myocardium and scar size. For each animal, three representative sections were quantified. Quantification bar graphs were generated *via* Prism 9.

### 4.13 Proteomic analysis of cytokine expression

A Proteome Profiler Rat Cytokine Array kit (R&D Systems, Minneapolis, MN, USA) was applied to assess the levels of inflammatory cytokines in rat serum collected four weeks after treatment. Briefly, the kit-provided Array Buffer 6 (block buffer) was used to block the array membranes for 1 h. Then, serum samples from 2 to 3 animals in each group were combined, mixed with Array Buffer 6, and then incubated with a reconstituted detection antibody cocktail for 1 h at room temperature. Subsequently, the blocked membranes were incubated with the prepared sample mixture overnight at 2–8 °C on a rocking platform shaker. The next day, following three washes, the membrane was incubated with streptavidin-HRP for 30 min at room temperature. Lastly, Chemi Reagent Mix was uniformly applied to each membrane, and images were captured using the chemiluminescence system utilized in the Western blotting analysis. The average density of each pair of dots was calculated and analyzed using ImageJ software (Version 1.0). A representative heat map and bar graph were generated *via* Prism 9.

### 4.14 Immunohistochemistry assessment

Heart cryosections were fixed with 4 % paraformaldehyde in PBS for 30 min, permeabilized and blocked with Protein Block Solution (DAKO) containing 0.1 % saponin for 1 h at room temperature. For immunostaining, the samples were incubated overnight at 4 °C with the following primary antibodies diluted in the blocking solution: mouse anti-rat α-SA (1:200, ab7817, Abcam) was used to identify cardiomyocytes; rat anti-mouse Ki67 antibody (1:200, ab15580, Abcam), and rabbit anti-mouse histone H3 phosphorylated at serine 10 (pH3, 1:200, ab5176, Abcam), were used to analyze cell-cycle re-entry, karyokinesis, and cytokinesis, respectively; rabbit anti-rat vWF (1:200, MA5-14029, Thermo Fisher Scientific) antibody was used to detect myocardial capillaries in the peri-infarct regions; goat anti-mouse Nkx2.5 (1:200, ab106923, Abcam) and rabbit anti-mouse Flk-1 (1:200, MA5-15157, Thermo Fisher Scientific) antibodies were used to examine endothelial progenitor cell recruitment; rabbit anti-mouse CD3 (1:200, ab16669, Abcam), and Mouse anti-rat CD68 (1:200, ab955, Abcam) antibodies were used to detect immune response. After three 10-min washes with PBS, samples were stained for 1.5 h at room temperature with fluorescent secondary antibodies including goat anti-rabbit IgG-Alexa Fluor 594 conjugate (1:400, ab150080, Abcam), goat anti-rat IgG-Alexa Fluor 488 conjugate (1:400, ab150157, Abcam), donkey anti-rabbit IgG-Alexa Fluor 488 conjugate (1:400, ab150073, Abcam), donkey anti-goat IgG-Alexa Fluor 594 conjugate (1:400, ab150136, Abcam), donkey anti-sheep IgG-Alexa Fluor 488 conjugate (1:400, ab150177, Abcam), goat anti-rabbit IgG-Alexa Fluor 488 conjugate (1:400, ab150077, Abcam), and goat anti-rabbit IgG-Alexa Fluor 594 conjugate (1:400, ab150080, Abcam), and goat anti-rat IgG-Cy5 conjugate (1:400, ab6563, Abcam) based on the isotopes of the primary antibodies. This was followed by 10 min of 4, 6-diamidino-2-phenylindole dihydrochloride (DAPI) staining for nucleus visualization. Slides were mounted with ProLong Gold mountant (Thermo Fisher Scientific) and viewed under a Zeiss LSM 710 confocal microscope (Carl Zeiss). Images were analyzed using NIH ImageJ software. Quantification bar graphs were generated *via* Prism 9.

### 4.15 Scanning electron microscopy (SEM)

The morphology and surface characteristics of the microneedles were examined using scanning electron microscopy (SEM). Briefly, bare PLGA nanoparticles (NPs) and membrane-coated PLGA NPs (S-NPs, P-NPs and SPIN) were resuspended in deionized (DI) water at a concentration of approximately 1 × 10⁸ particles/mL using probe sonication (30 W, 20 s) to ensure uniform dispersion. A 10 μL aliquot of each suspension was dropped onto a clean glass coverslip and allowed to air-dry under a laminar flow hood. The dried samples were sputter-coated with a thin layer of gold/platinum (Au/Pt) and imaged using a scanning electron microscope (SEM, Hitachi S-4800, Japan).

### 4.16 Statistics

All experiments were conducted independently a minimum of three times, with results displayed as mean ± SD. To compare two groups, a two-tailed unpaired Student's t-test was utilized. For comparisons involving more than two groups, one-way ANOVA followed by a post-hoc Bonferroni test and two-way ANOVA followed by a post-hoc Bonferroni test were utilized. Single, double, and triple asterisks denote p < 0.05, 0.01, and 0.001, respectively; p < 0.05 was regarded as statistically significant.

## CRediT authorship contribution statement

**Mingqian He:** Writing – original draft, Visualization, Validation, Project administration, Methodology, Formal analysis, Data curation, Conceptualization. **Yuan Li:** Writing – original draft, Validation, Formal analysis, Data curation. **Dashuai Zhu:** Validation, Investigation, Formal analysis, Data curation. **Junlang Li:** Formal analysis, Data curation. **Meggie Cangu:** Formal analysis. **Panagiotis Tasoudis:** Formal analysis, Data curation. **Jiazhu Xu:** Formal analysis, Data curation. **Thomas G. Caranasos:** Conceptualization. **Yi Hong:** Supervision. **Ke Huang:** Writing – review & editing, Supervision, Funding acquisition.

## Ethics approval and consent to participate

All animal procedures were conducted in accordance with the guidelines and regulations approved by the Institutional Animal Care and Use Committee (IACUC) of the University of North Carolina at Chapel Hill and North Carolina State University (approval number: 22-422-B). The study adhered to the principles outlined in the Guide for the Care and Use of Laboratory Animals published by the National Institutes of Health. All efforts were made to minimize animal suffering and to use the minimum number of animals necessary to obtain statistically significant data.

## Declaration of competing interests

Yi Hong is an editorial board member for Bioactive Materials and was not involved in the editorial review or the decision to publish this article. All authors declare that there are no competing interests.
