# Supplementary material for: Stromal-platelet membrane-inspired nanoparticles (SPIN) for targeted heart repair
Source: Bioact Mater. 2025 Jul 7;53:45–57. doi: 10.1016/j.bioactmat.2025.06.055 (PMC12272480; doi:10.1016/j.bioactmat.2025.06.055)
Supplement: Multimedia component 1 [file mmc1.docx]

**Supplementary Data**


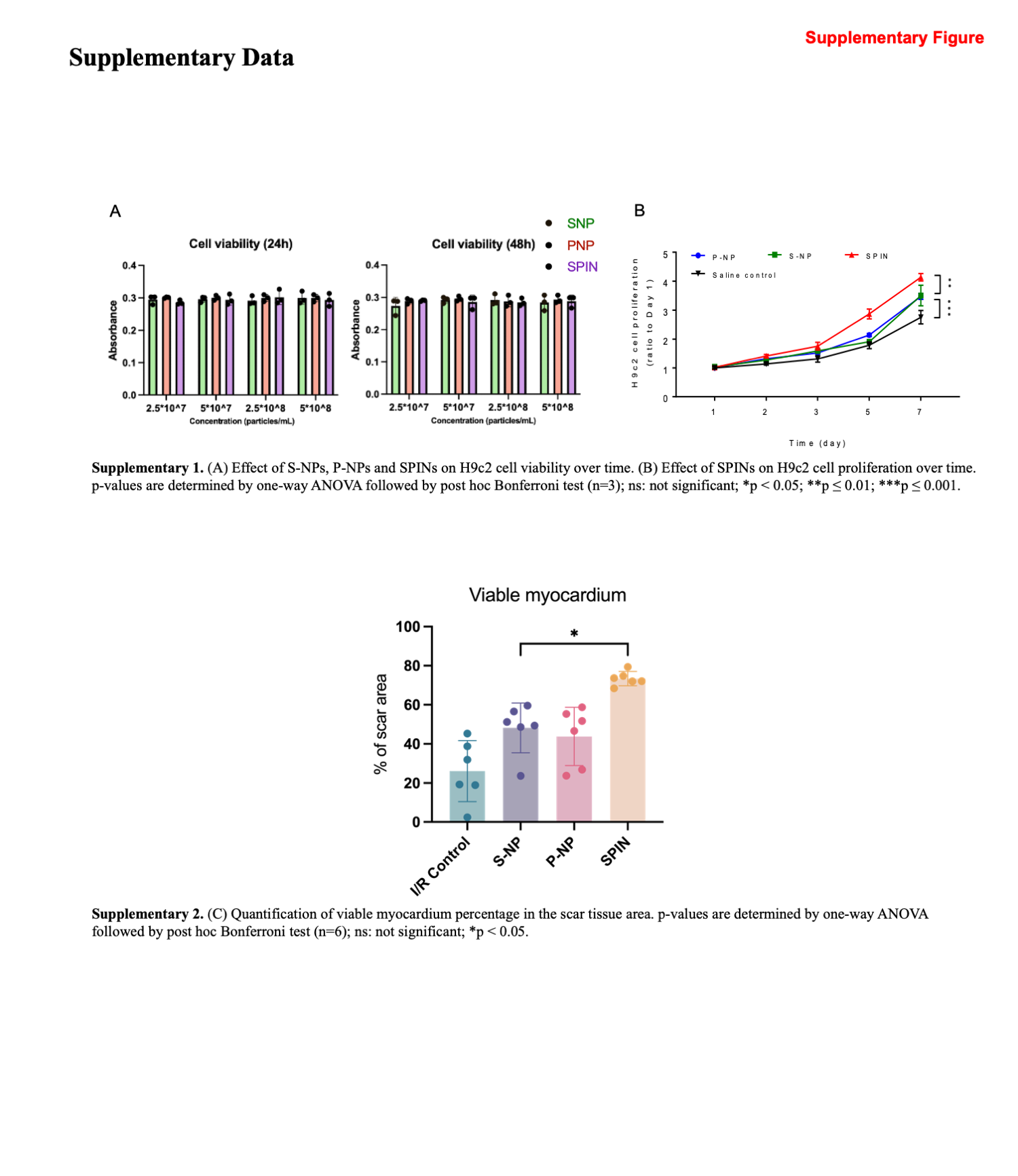


**Supplementary 1.** (A) Effect of S-NPs, P-NPs and SPINs on H9c2 cell viability over time. (B) Effect of SPINs on H9c2 cell proliferation over time. p-values are determined by one-way ANOVA followed by post hoc Bonferroni test (n=3); ns: not significant; **P* < 0.05; ***P* ≤ 0.01; ****P* ≤ 0.001.


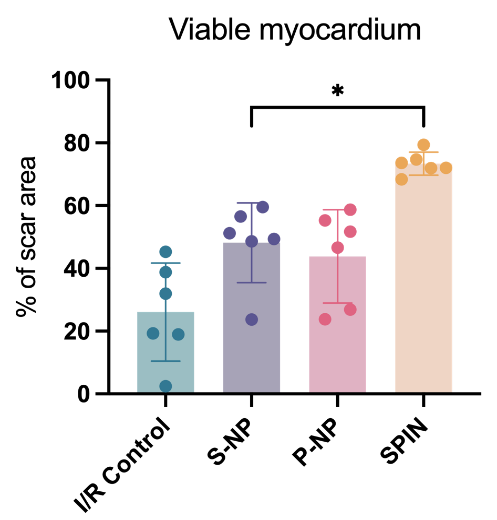


**Supplementary 2.** (C) Quantification of viable myocardium percentage in the scar tissue area. p-values are determined by one-way ANOVA followed by post hoc Bonferroni test (n=6); ns: not significant; *p < 0.05.


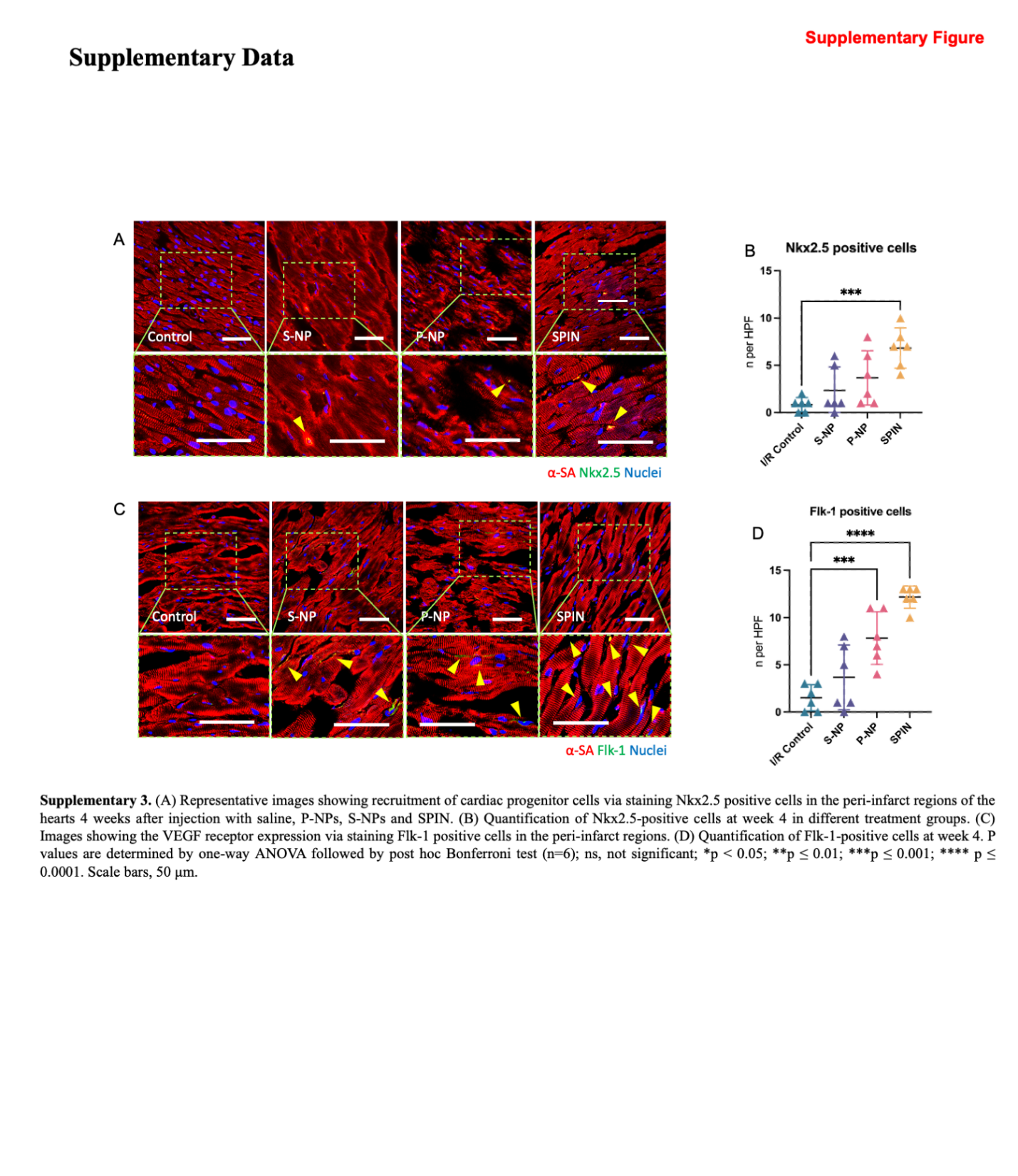


**Supplementary 3.** (A) Representative images showing recruitment of cardiac progenitor cells via staining Nkx2.5 positive cells in the peri-infarct regions of the hearts 4 weeks after injection with saline, P-NPs, S-NPs and SPIN. (B) Quantification of Nkx2.5-positive cells at week 4 in different treatment groups. (C) Images showing the VEGF receptor expression via staining Flk-1 positive cells in the peri-infarct regions. (D) Quantification of Flk-1-positive cells at week 4. P values are determined by one-way ANOVA followed by post hoc Bonferroni test (n=6); ns, not significant; **P* < 0.05; ***P* ≤ 0.01; ****P* ≤ 0.001; **** *P* ≤ 0.0001. Scale bars, 50 μm.
